# Evidence against a role for jaagsiekte sheep retrovirus in human lung cancer

**DOI:** 10.1186/s12977-017-0329-6

**Published:** 2017-01-20

**Authors:** A. Dusty Miller, Marcelo De las Heras, Jingyou Yu, Fushun Zhang, Shan-Lu Liu, Andrew E. Vaughan, Thomas L. Vaughan, Raul Rosadio, Stefano Rocca, Giuseppe Palmieri, James J. Goedert, Junya Fujimoto, Ignacio I. Wistuba

**Affiliations:** 1 Fred Hutchinson Cancer Research Center, Seattle, WA USA; 2 Department of Pathology, University of Washington, Seattle, WA USA; 3 Department of Animal Pathology, Zaragoza University, Zaragoza, Spain; 4 Center for Retrovirus Research, Department of Veterinary Biosciences, The Ohio State University, Columbus, OH USA; 5 Department of Molecular Microbiology and Immunology, Bond Life Sciences Canter, University of Missouri, Columbia, MO USA; 6 Department of Medicine, University of California San Francisco, San Francisco, CA USA; 7 Program in Epidemiology, Fred Hutchinson Cancer Research Center, Seattle, WA USA; 8 Veterinary Faculty, National University of San Marcos, Lima, Peru; 9 Department of Veterinary Medicine, Sassari University, Sassari, Italy; 10 Unit of Cancer Genetics, Institute of Biomolecular Chemistry, National Research Council, Sassari, Italy; 11 Division of Cancer Epidemiology and Genetics, National Cancer Institute, Rockville, MD USA; 12 Department of Translational Molecular Pathology, University of Texas MD Anderson Cancer Center, Houston, TX USA; 13 17915 Edmundson Rd, Sisters, OR 97759 USA

**Keywords:** Jaagsiekte sheep retrovirus, Human lung cancer, Ovine pulmonary adenocarcinoma

## Abstract

**Background:**

Jaagsiekte sheep retrovirus (JSRV) causes a contagious lung cancer in sheep and goats that can be transmitted by aerosols produced by infected animals. Virus entry into cells is initiated by binding of the viral envelope (Env) protein to a specific cell-surface receptor, Hyal2. Unlike almost all other retroviruses, the JSRV Env protein is also a potent oncoprotein and is responsible for lung cancer in animals. Of concern, Hyal2 is a functional receptor for JSRV in humans.

**Results:**

We show here that JSRV is fully capable of infecting human cells, as measured by its reverse transcription and persistence in the DNA of cultured human cells. Several studies have indicated a role for JSRV in human lung cancer while other studies dispute these results. To further investigate the role of JSRV in human lung cancer, we used highly-specific mouse monoclonal antibodies and a rabbit polyclonal antiserum against JSRV Env to test for JSRV expression in human lung cancer. JSRV Env expression was undetectable in lung cancers from 128 human subjects, including 73 cases of bronchioalveolar carcinoma (BAC; currently reclassified as lung invasive adenocarcinoma with a predominant lepidic component), a lung cancer with histology similar to that found in JSRV-infected sheep. The BAC samples included 8 JSRV DNA-positive samples from subjects residing in Sardinia, Italy, where sheep farming is prevalent and JSRV is present. We also tested for neutralizing antibodies in sera from 138 Peruvians living in an area where sheep farming is prevalent and JSRV is present, 24 of whom were directly exposed to sheep, and found none.

**Conclusions:**

We conclude that while JSRV can infect human cells, JSRV plays little if any role in human lung cancer.

## Background

Jaagsiekte sheep retrovirus (JSRV) causes a contagious lung cancer in sheep called ovine pulmonary adenocarcinoma (OPA). Virus is secreted into lung fluid that can be aerosolized by coughing or sneezing allowing virus transmission to other animals [1]. The disturbing finding that human cells express a functional receptor for JSRV entry [2, 3], and histologic similarities between OPA and human lung cancers, including bronchioalveolar carcinoma (BAC) and other adenocarcinomas, has led to a search for a possible role for JSRV or related viruses in human lung cancer [4–12]. Of further concern, the envelope (Env) protein of JSRV is a potent oncoprotein and expression of Env alone in lungs of mice and sheep results in disease similar to that caused by the intact virus in sheep [13, 14]. Thus, JSRV is fully competent to induce lung cancer without the need for extensive virus replication and insertional activation of cellular oncogenes, as is seen in other retroviral cancers such as those induced by the murine leukemia retroviruses.

Several studies have addressed the possible role of JSRV in human lung cancer with mixed results. In an extensive initial study of human lung cancer specimens from around the world, De las Heras et al. [4] used a rabbit polyclonal antiserum against JSRV Gag proteins to perform an immunohistochemical analysis of formalin-fixed paraffin-embedded lung tumor tissue. The authors found positive cytoplasmic staining of tumor cells in 30% of 129 BAC specimens, 26% of 65 other adenocarcinomas, and 2 of 7 large cell carcinomas. No staining was observed for 21 nontumor lung lesions, four normal lung tissues, 23 adenocarcinomas from other organs and a cell line expressing a human endogenous retrovirus. These results suggested widespread involvement of JSRV in human lung cancer. However, two other groups found no evidence for JSRV by PCR of DNA or RNA extracted from similar formalin-fixed paraffin-embedded lung tumor specimens [5, 6]. Samples included 26 BAC and 25 other adenocarcinoma specimens in one study [5] and 18 BAC and 6 other lung tumors in the other [6]. Both of these studies had reasonable positive controls and controls to ensure the quality of the DNA extracted from the fixed tissue. In addition, the positive results in the initial study [4] were called into question by additional reports indicating that the polyclonal antiserum used in the initial study showed cross-reactivity with an endogenous human retroviral protein, leading to false positive results [7], and that no additional evidence of retroviral infection could be found in the lung cancer specimens that stained positive by the JSRV Gag antiserum [8].

In other positive studies, Morozov et al. [9] detected JSRV DNA by nested PCR in blood of Africans from Nigeria and Cameroon, but not in a set of only 4 BAC specimens from patients from Russia and Germany. In the case of the blood samples, 28% were positive using one primer set, 11% using a second set, and 0% using a third primer set, indicative of technical issues in JSRV detection. Rocca et al. [10] reported that JSRV was frequently found in paraffin sections of BAC specimens from lung cancer patients in Sardinia (91% positive), where sheep farming is prevalent and the sheep are known to be infected with JSRV, but was infrequently found in patients with BAC from the Campania region (10% positive), where sheep farming is absent. JSRV was detected by nested PCR of JSRV gag DNA sequences in formalin-fixed paraffin-embedded tissues. However, 54% of normal lung specimens from Sardinia were also positive for JSRV DNA, showing that the presence of JSRV DNA was not well correlated with lung cancer. A more recent study found evidence for JSRV Env expression in human tissue arrays by immunostaining with JSRV Env monoclonal antibodies (Mabs) and by PCR analysis for JSRV *gag* and *env* DNA [11]. Unfortunately, original tumor samples were not available to definitively corroborate these results, and the interpretation of these results is problematic (see “Discussion”). Most recently, a high-throughput sequencing approach found no evidence for JSRV in five human lung adenocarcinomas [12]. While this would provide a definitive method for detecting JSRV involvement in human lung cancer, the high cost of this approach limits its application to small numbers of lung cancer subjects.

To help resolve the role of JSRV in human lung cancer, we used highly specific mouse monoclonal antibodies (Mab) for immunohistochemical detection of the JSRV Env protein in fixed tissue sections [15], reasoning that detection of this viral oncoprotein in human lung cancer would be a sensitive and accurate indicator of a biological role for JSRV in human lung cancer. As shown previously, a mixture of two anti JSRV Env Mabs could readily detect JSRV in lungs of sheep from the USA, Peru, Spain, Kenya, and South Africa, showing that these Mabs could detect many wild strains of JSRV [15]. Human lung cancer samples analyzed in the current study include BAC and other lung cancer specimens, mostly from the United States, and Sardinian lung cancer samples that tested positive for JSRV DNA by nested PCR [10]. All of the lung cancer samples tested negative for JSRV Env expression by immunohistochemistry using the Mabs or a rabbit anti-JSRV Env antiserum. Furthermore, we show that JSRV virus is able to infect cultured human cells, and that our Mabs could detect JSRV Env expression in human cells if present. Nor could we find virus-neutralizing antibodies directed against the JSRV Env protein in 138 human subjects from the Central Sierra region of Peru who live in an area where sheep infected with JSRV are common. Together our results indicate that JSRV plays little if any role in human lung cancer.

## Results

### Lack of JSRV Env expression in lung cancer specimens from the United States

A set of 53 BAC and 50 other lung cancer samples from lung cancer patients seen at the MD Anderson Cancer Center were analyzed for expression of JSRV Gag and Env proteins by Mab and polyclonal rabbit antiserum staining of fixed tumor-bearing lung tissue [Table 1; BAC samples 1–53 and other lung cancer (LCA) samples 1–50]. We focused on analysis of BAC samples because this histological classification is thought to most closely resemble the type of cancer seen in sheep infected with JSRV. Note that the currently accepted lung cancer classification that most closely corresponds to BAC is lung invasive adenocarcinoma with a predominant lepidic component with and without mucinous features [16, 17]. However, because the lung cancer samples analyzed here were collected and histologically classified before this revision, we have kept the older terminology. The human lung cancers arose in patients 33–84 years of age, of both sexes, of White, Black, and Hispanic ethnicities, and who currently smoked, formerly smoked, or who never smoked. All of the human lung cancer specimens were completely negative for JSRV Env and SU (the extracellular domain of the Env protein) staining performed by using a mixture of two highly-specific anti-JSRV-Env mouse Mabs or a rabbit SU (extracellular domain of Env) antiserum (Table 1). In contrast, lung cancer specimens from sheep infected with JSRV and from mice exposed to an AAV vector that expresses JSRV Env were accompanied by readily detectable JSRV Env expression. Because the Env protein of JSRV is the sole active oncogene in JSRV, these findings indicate that JSRV was not responsible for the lung cancers in these patients.

**Table 1:**
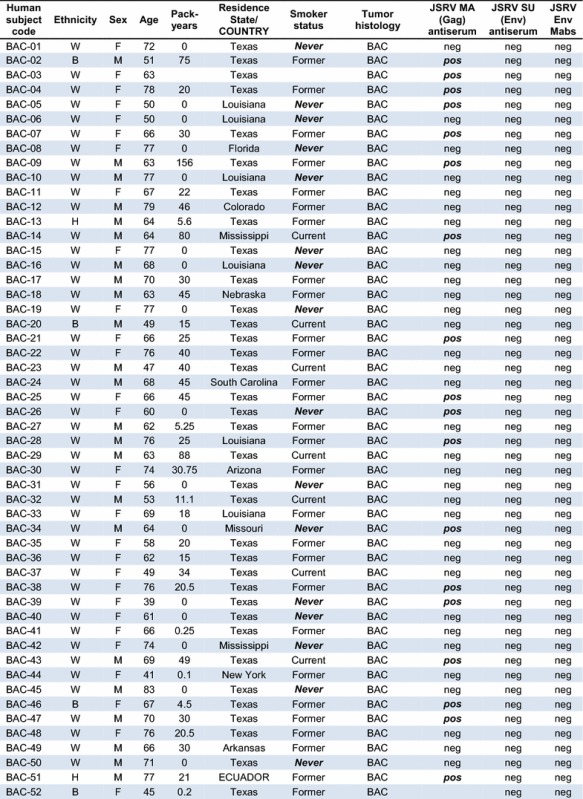

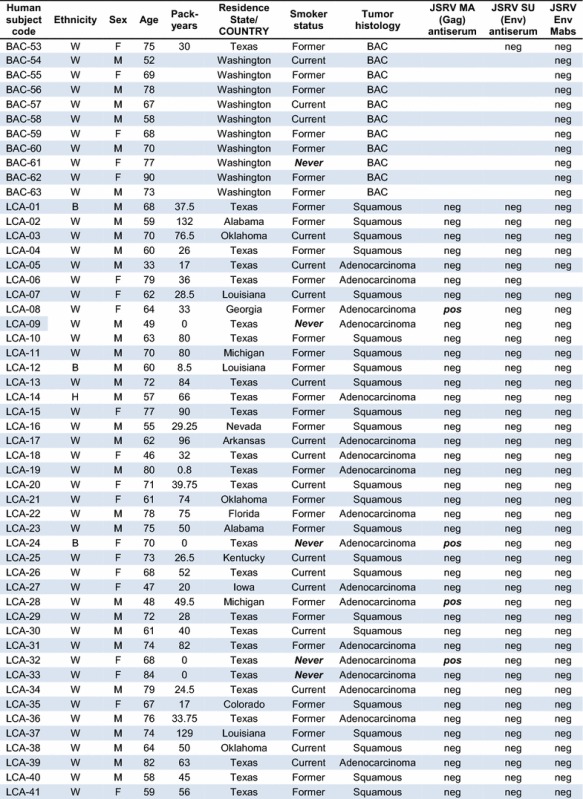

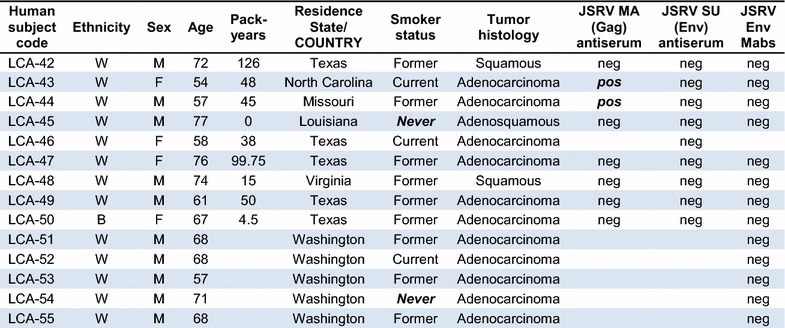
Epidemiologic data and JSRV immunohistochemistry results for lung cancer patients seen at MD Anderson and Fred Hutchinson Cancer Centers

Lung cancer samples obtained at the MD Anderson Cancer Center (BAC-1 to 53 and LCA-1 to 50) were collected between January 1986 and December 2004, and those at the Fred Hutchinson Cancer Research Center (BAC-54 to 63 and LCA-51 to 55) were collected between October 1994 and February 1999. Ethnicity: *W* White, *B* Black, *H* Hispanic; *Pack years*, number of packs of cigarettes smoked per day × number of years the person has smoked, *neg* negative, *pos* positive. *Never* smoker status and positive JSRV MA (Gag) staining are highlighted in **bolditalic**. Absence of an entry in the results columns indicate the test was not performed

In contrast, we observed positive JSRV Gag matrix (MA) protein staining of many BAC samples (18 of 51 = 35%, Table 1) and some LCA samples (6 of 49 = 12%, Table 1), similar to the initial study showing a relatively high incidence of JSRV Gag expression (30%) in lung cancer specimens from around the world [4]. We interpret these data as indicating spurious staining by the JSRV Gag MA protein antiserum, as previously described [7]. Importantly, none of these Gag-positive samples stained positive when using the anti-JSRV Env Mabs or the rabbit anti-JSRV Env polyclonal antiserum.

We also tested for JSRV Env expression in a set of 10 BAC and 5 other adenocarcinoma samples from newly-diagnosed patients participating in a Fred Hutchinson Cancer Research Center study (Table 1; BAC samples 54–63 and LCA samples 51–55). These lung cancer specimens were freshly frozen, sectioned in a cryostat, and the sections were placed on slides and fixed using paraformaldehyde in phosphate-buffered saline. All of the 10 BAC and 5 adenocarcinoma specimens were completely negative for JSRV Env Mab staining, while control positive sheep and mouse tumors stained in parallel were strongly positive. Again, because the Env protein of JSRV is the active oncogene in JSRV, these findings indicate that JSRV played no role in the lung cancers of these patients.

### Lack of JSRV Env expression in lung cancer samples from Sardinia

**Fig. 1:**
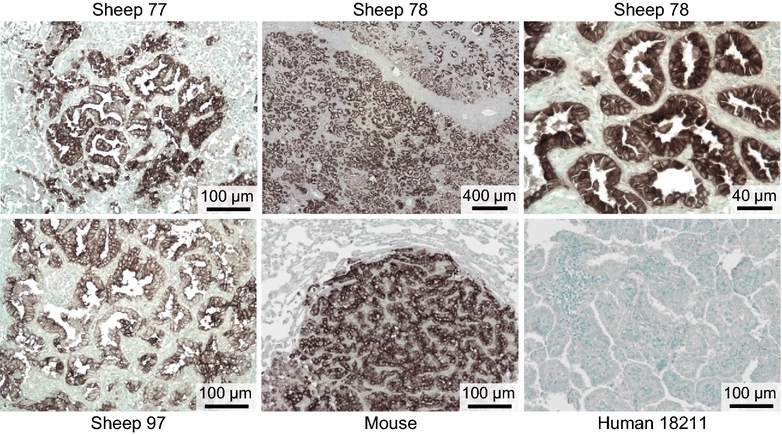
Histological analysis of lung tissue from three Sardinian sheep with OPA, from a mouse with JSRV Env-induced lung tumors, and from a representative Sardinian lung cancer patient. Histological staining, as described in “Methods”, was performed at the same time for all tissues shown. *Scale bars* are shown at the *bottom of each panel*

Ten of eleven (91%) lung cancer (BAC) specimens from patients living on the Italian island of Sardinia, where sheep farming is prevalent and the sheep are known to be infected with JSRV, were previously found positive for JSRV DNA by nested PCR, suggesting a role for JSRV in lung cancer in this region [10]. In contrast, only 1 of 10 (10%) of BAC specimens from patients living in the Campania region of Italy, where sheep farming is absent, were found positive for JSRV DNA [10]. We analyzed BAC specimens from eight JSRV PCR-positive and two JSRV PCR-negative Sardinians, and OPA specimens from 3 Sardinian sheep, for JSRV Env expression. Histologic staining of all samples was performed in parallel to control for any variables in the staining procedure. The tumor specimens were formaldehyde fixed and paraffin embedded. All of the human lung cancer specimens were completely negative for Env Mab staining while all three of the sheep samples were strongly positive (see Fig. 1 for images of the three sheep samples and one representative human tumor sample), indicating a lack of JSRV involvement in the human lung cancer etiology despite the JSRV-positive PCR results.

### JSRV Env expression in cultured human cells can be detected by Mab staining

**Fig. 2:**
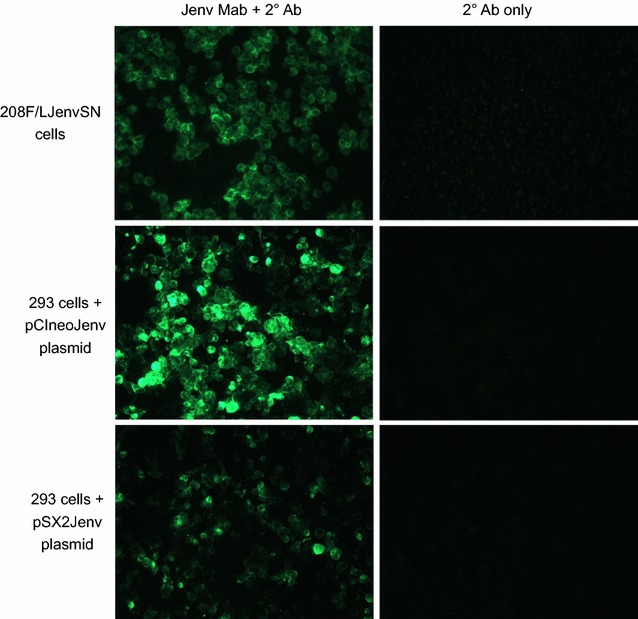
JSRV Env Mabs bind to human 293 cells expressing the JSRV Env protein. Human 293 cells were transiently transfected with the JSRV Env-expression plasmids pCIneoJenv, which contains the JSRV Env coding region cloned into the pCIneo expression plasmid (Promega, Madison, WI), or pSX2Jenv [2]. JSRV Env expression is driven by a CMV promoter in pCIneoJenv, and by a Moloney murine leukemia virus (MoMLV) promoter in pSX2Jenv. The 208F/LJenvSN cells are 208F rat fibroblasts transduced with the LJenvSN retroviral vector, which contains the JSRV Env coding region cloned into the LXSN retroviral vector [41], and Env is expressed from the MoMLV promoter in LXSN. For analysis, the adherent cells were suspended using EDTA (no trypsin), centrifuged onto slides, fixed with formaldehyde, and subjected to immunostaining. The secondary (2°) antibody was a goat anti-mouse IgG (H + L) conjugated to Alexa Fluor 488 (a green-fluorescent dye) obtained from Invitrogen. Env expression is detected in all of these cell types by the JSRV Env Mabs

We considered the possibility that the JSRV Env Mabs might not detect JSRV Env expression in human cells because of human-specific protein modifications that might interfere with Mab binding. To test for this possibility, human 293 cells were transfected with plasmids that expressed JSRV Env by using either a CMV promoter (pCIneoJenv) or an SV40 promoter (pSX2Jenv). Next, immunostaining was performed using the JSRV Env Mabs followed by a secondary antibody conjugated to the green dye, Alexa Fluor 488. As a positive control, rat 208F fibroblasts that stably express JSRV Env were analyzed in parallel. The human 293 cells transfected with either JSRV Env expression plasmid exhibited bright but heterogeneous immunostaining, typical of transiently transfected cells, which was not seen when only the secondary antibody was used (Fig. 2). The JSRV-Env-expressing rat cells showed a more homogeneous staining pattern (Fig. 2), typical of stably transduced cells. These results show that the JSRV Env Mab is capable of detecting JSRV Env expression in human cells.

### JSRV virus can infect human cells

**Fig. 3:**
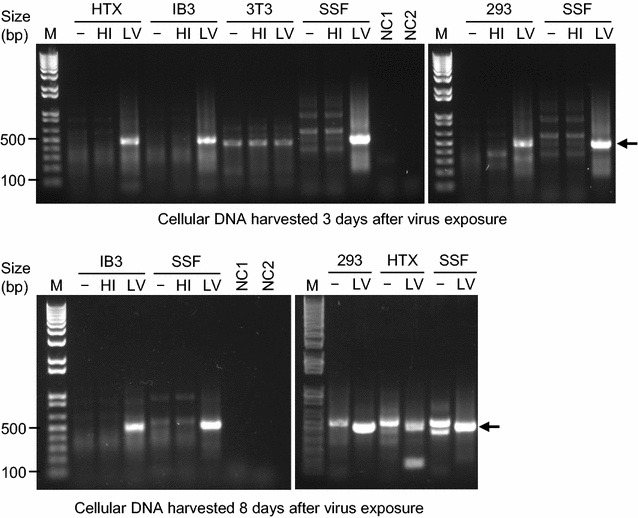
PCR detection of JSRV sequences in the genomic DNA of cells exposed to live JSRV virus (LV), heat-inactivated virus (HI) or culture medium alone (−). PCR amplification procedures are described in “Methods”. M indicates a 1 kb Plus DNA Ladder (Thermo Fisher) with select band sizes indicated. The expected JSRV amplification product (*arrows*) has a size of 502 bp, which is clearly visible in the live virus (LV) lanes of all cell types except for the NIH 3T3 cells, which lack a functional cell-surface receptor for JSRV. This band is absent in all of the heat inactivated (HI) virus and negative control (−) lanes. The *top two panels* show an analysis of cellular DNA harvested 3 days after virus exposure, and this experiment was repeated once with identical results. The *bottom two panels* show an analysis of cellular DNA harvested 8 days after virus exposure, and this experiment was performed once

It is known that JSRV Env can mediate entry of retroviral vectors into human cells [2, 3]. Entry is mediated by virus binding to the cell-surface protein Hyal2, which is a functional receptor for retroviral vectors bearing JSRV Env in sheep and human cells, but not in mouse cells for example [2]. However, it is not known whether there are post-entry restrictions to JSRV infection in human cells that might block virus infection, and if so, might explain the absence of JSRV Env expression in human lung cancer. To address this issue, we generated infectious JSRV virus and tested for virus entry and conversion of the viral RNA genome into DNA in SSF sheep skin fibroblasts and in several human cell lines, including the human bronchial epithelial cell line IB3 [18]. IB3 cells were made by immortalization of primary human bronchial epithelial cells by expression of SV40 T antigen, and retain many of the biochemical characteristics of normal lung epithelial cells [18], and thus have characteristics more like the natural lung epithelial cell targets for JSRV oncogenesis. JSRV DNA was present in DNA extracted from the sheep fibroblasts and all of the human cell lines exposed to live JSRV virus, but was not present in DNA of sheep or human cells exposed to heat-inactivated virus or to culture medium alone (Fig. 3). JSRV DNA was not found in NIH 3T3 mouse cells exposed to live JSRV virus (Fig. 3), as expected because these cells do not express a functional cell-entry receptor for JSRV. Viral DNA was detected in sheep and in all of the human cells at 3 and 8 days after exposure of the cells to live JSRV virus, showing that viral DNA persisted during culture of the cells, and indicating but not proving that the virus was integrated into cellular DNA. Together, these results show that JSRV is able to infect human cells, as measured by persistence of JSRV DNA in the genomic DNA of several cultured human cell lines exposed to live JSRV virus, and indicate that a human-specific block to infection is not responsible for the absence of JSRV infection in human lung cancer patients.

### Lack of neutralizing antibodies against JSRV in Peruvians living in areas coinhabited by JSRV infected sheep

Immunodeficient mice receiving an intranasal administration of a viral vector that expresses the JSRV Env protein rapidly died from widespread JSRV Env-induced lung cancer, while immunocompetent mice subjected to the same treatment rarely developed tumors [13]. The immunocompetent mice developed high-titer antibodies against the JSRV Env protein that were capable of neutralizing a viral vector coated with JSRV Env [13], and that presumably played a role in clearance of JSRV Env-expressing lung tumor cells. To explain our inability to detect JSRV Env expression in human lung cancer, we hypothesized that JSRV might be capable of infecting humans, but that the resulting immune response clears infected lung cells and prevents lung cancer initiation and/or progression.

To address this possibility, we tested for such immune responses in 138 men, women and children from the Central Sierra region of Peru who live in an area where sheep infected with JSRV are common (~2% annual mortality and 4–6% morbidity from JSRV infection), and 24 of whom were involved in occupations that involved direct exposure to sheep (sheep herders, n = 6; veterinary technicians, n = 6; sheep farmers, n = 4; veterinarians, n = 4; and slaughterhouse workers, n = 4). We tested for anti-JSRV Env neutralizing antibodies by measuring inactivation of a retroviral vector bearing the JSRV Env protein (LAPSN(PJ4) [2]), which is on the surface of JSRV virions, and is the protein responsible for oncogenic transformation of infected cells. The human serum samples were mixed with the LAPSN(PJ4) vector, which encodes human placental alkaline phosphatase, and the titer of the vector was measured using human HTX cells as targets (see “Methods”). 135 of the 138 Peruvian serum samples we tested were unable to inactivate the JSRV vector (antibody titer <2), while sera from an immunocompetent mouse exposed to an AAV vector encoding JSRV Env [13] inactivated the vector (titer >200). Three of the Peruvian serum samples showed titers between 3 and 30. Because it has been reported that human complement can neutralize some retroviruses in the absence of virus-specific antibodies [19–21], we next tested for virus inactivation by these three serum samples after heat-inactivation of the sera (56 °C for 30 min) to destroy complement activity. Heat-inactivation reduced the neutralizing activity of these three serum samples to undetectable levels (titer <2). In contrast, heat inactivation of sera from four sheep infected with JSRV and from one immunocompetent mouse that had received an intranasal administration of a vector encoding JSRV Env had no effect on the neutralizing activity of these sera (data not shown). In conclusion, we were unable to detect a virus-neutralizing antibody response directed against JSRV Env in any of these Peruvian subjects, indicating a lack of JSRV infection of these subjects.

## Discussion

JSRV utilizes the cell-surface protein Hyal2 to gain entry into cells [3]. A functional Hyal2 receptor is expressed on sheep, human, monkey, canine, bovine, and rabbit cells, but not on mouse, rat or hamster cells [2]. Here we show for the first time that the JSRV RNA genome can be reverse transcribed into DNA and persist in human cells, indicating the absence of restriction factors that might block JSRV infection at a post-entry step in virus infection. Furthermore, the JSRV Env protein alone can transform a variety of cultured cell types [3, 22–24] and induce lung cancer in mice [13] and sheep [14]. Note that Hyal2 is not required for oncogenic transformation by JSRV, because lung tumors can be induced by expression of JSRV Env alone in mice, which express a Hyal2 protein that does not bind JSRV Env and cannot mediate JSRV entry into cells [13, 15]. However, we find no evidence for JSRV involvement in human lung cancer.

We do know that while expression of JSRV Env in lungs of immunodeficient Rag2-knockout mice that lack mature T and B cells can cause rapid development of tumors and death, immunocompetent mice are resistant to lung cancer induction by the same treatment [13] (although this result appears to be dependent of the strength of the promoter that drives JSRV Env expression [25]). In contrast, the sheep genome contains many endogenous copies of retroviral elements related to JSRV, and sheep are relatively immunotolerant of JSRV presumably because of expression of proteins related to those of JSRV by these endogenous elements [26]. These considerations suggest that the best place to look for possible JSRV involvement in human lung cancer would be in lung cancer patients who have had extensive exposure to JSRV-infected sheep and who are immunodeficient because of genetic or environmental factors, including infection by immunosuppressive agents such as HIV. For the present, however, it appears that JSRV plays little if any role in lung cancer in humans.

Initially, we were very excited to test for expression of JSRV Env protein in human lung cancer specimens from Sardinia, Italy, where sheep farming is prevalent, JSRV is present in these sheep, and PCR analysis indicated the presence of JSRV in the human lung cancer specimens from Sardinia but not from Campania where sheep farming is absent [10]. However, we find no evidence for JSRV Env expression in the Sardinian lung cancer samples. To understand the discrepancy between the positive PCR results and our negative Mab staining results, we note several issues with the PCR analysis and its interpretation. First, the authors state that the PCR primers they used were specific for endogenous JSRV, which is present in multiple copies in sheep. But the acknowledged source of these primer sequences was a report from Cousens et al., in which the JSRV primers used were specifically designed to not amplify sheep endogenous DNA sequences [27]. Indeed, National Center for Biotechnology Information BLAST analysis (performed 17 December 2016) of the JSRV sequence identified by Rocca et al. in human specimens [10] most closely matches exogenous JSRV sequences. Thus it is a surprise that normal liver and normal lung samples from Sardinians not suffering from lung cancer were also PCR-positive for JSRV. The authors then went on to argue that these results suggested the presence of JSRV as an endogenous virus present in the genomes of the Sardinian people. We think this possibility is very unlikely (but could be easily tested by genomic sequencing, for example), and instead suggest either the PCR results represent false positives, or that the presence of JSRV-infected sheep DNA in food or in the environment is responsible for the positive PCR results. We believe the negative JSRV-Env Mab and rabbit antiserum staining definitively rules out a role for JSRV in the Sardinian lung cancer samples analyzed here.

Previous reports have shown that human serum can inactivate a variety of retroviruses, including Moloney murine leukemia virus, feline leukemia virus, and New Zealand black mouse xenotropic murine leukemia virus [19–21]. While the initial studies indicated that the viruses were lysed by complement in the absence of antibody involvement, later studies have indicated that natural antibodies against the Galα1–3Gal sugar epitope present on cell-surface proteins of all mammals, with the exception of old-world primates (including humans), are required for complement lysis of various retroviruses [28, 29]. Regardless, the virus neutralizing activity in human serum has been proposed as a natural barrier to human infection by retroviruses and other viruses present in animals. Thus, it is surprising that most of the Peruvian serum samples we tested (98%) were unable to neutralize the retroviral vector bearing the JSRV Env protein, even in the absence of heat treatment of the sera to inactivate complement. The LAPSN(PJ4) vector used in the neutralization assay was produced from mouse retroviral packaging cells [2], and the Galα1–3Gal sugar epitope is added to cell-surface proteins made by mouse cells. Previously, it has been shown that the JSRV Env protein stabilizes retroviral vectors against lung surfactant, centrifugation, and freeze–thaw cycling compared to other retroviral Env proteins [30]. These considerations suggest that JSRV is more resistant than other animal retroviruses to antiviral restrictions in humans that operate prior to virus entry into cells.

In contrast to results reported here, others have claimed evidence for the presence of JSRV in human lung cancer as determined by using the same anti-JSRV Env Mabs we used here [11]. We disagree with this conclusion based primarily on the data presented in Fig. 1 of that report, which purports to show positive Mab staining of human lung cancer tissue. We argue that none of the human tissue staining presented in Fig. 1 closely resembles the Mab staining shown for JSRV-induced sheep tumors in panel R of that Figure. In our experience, the mixture of anti-JSRV Env Mabs that we used here provides robust staining of JSRV-induced tumors in sheep and JSRV Env-induced tumors in mice, and allows single tumor cells to be easily identified ([15] and Fig. 1 of the current report). There is no need for a grading system for the staining results that was used in Ref. [11]. Indeed, the staining of the human lung cancer tissue sections [11] resembles background staining we occasionally see with polyclonal mouse anti-JSRV Env antibodies (data not shown). Secondly, although JSRV *env* and *gag* sequences were detected in some human tissue samples and in pooled tissue arrays, this data is unconvincing. Detection of trace amounts of specific DNA sequences by highly-sensitive PCR techniques is notorious for yielding false-positive results, especially when the testing lab or nearby labs already have plasmids or PCR amplification products bearing very high numbers of copies of the sequence to be detected.

Previous work has shown that the mixture of the two anti-JSRV Env Mabs used in the current report can readily detect lung cancer caused by JSRV in sheep from the USA, Peru, Spain, Kenya and South Africa [15], and in this report we demonstrate detection of JSRV in lung cancer of sheep from Sardinia, Italy (Fig. 1). The Mabs are also known to detect Env expression in tumors caused by a closely related virus, enzootic nasal tumor virus (ENTV) [15]. Immunohistochemical staining of JSRV-infected tumor cells by the Mabs is clear and unequivocal, and the Mabs provide an unlimited reagent for diagnostic testing of the involvement of JSRV and related viruses in lung and nasal tumors of sheep and goats. Here we show that these Mabs recognize JSRV Env expression on human cells as well, indicating that they will be useful in future studies of the possible involvement of JSRV and ENTV in human cancers.

## Methods

### Lung tumor specimens

Lung tumor specimens were obtained from stored material from previous Institutional Review Board-approved studies. The current studies were performed with Institutional Review Board approval from the Fred Hutchinson Cancer Research Center and the MD Anderson Cancer Center.

### Immunohistochemistry

Lung cancer specimens were fixed in 10% formalin or 2% paraformaldehyde in phosphate-buffered saline and were embedded in paraffin wax. The JSRV Env monoclonal antibodies (Mabs) used were an equal mixture of culture medium conditioned by the B3 and C9 mouse hybridoma cell lines [15]. For analysis, ~5 µm thick tissue sections were deparaffinized, subjected to antigen retrieval, stained with Mabs, and counterstained with methyl green as previously described [15]. Control JSRV-positive lung cancer specimens were obtained from sheep infected with JSRV or from mice with lung tumors induced by transfer and expression of JSRV Env to their lungs by using an AAV vector [13]. At least one positive control sample was analyzed in parallel with each batch of human lung cancer samples to be sure the immunohistochemical staining succeeded.

Lung cancer specimens were also stained using rabbit polyclonal antiserum against the surface domain (SU) of the JSRV Env protein (kindly provided by the Moredun Research Institute, Edinburgh, Scotland), and a polyclonal antiserum against the matrix domain (MA) of the JSRV Gag protein (kindly provided by Massimo Palmarini) obtained by immunizing rabbits with a recombinant protein consisting of the N-terminal domain of the JSRV Gag protein that was expressed in bacteria (Proteintech). Lung sections were deparaffinized, subjected to antigen retrieval with citrate buffer pH 6.0 in a pressure cooker, and stained following routine procedures as previously described [7, 31, 32]. Control JSRV-positive lung cancer specimens were obtained from sheep with naturally-acquired OPA. Control negative tissue sections were obtained from a dog with naturally-acquired lung adenocarcinoma. Pre-immune sera were collected before immunizations. Normal rabbit serum and immunohistochemistry buffer were used as negative controls for antiserum staining.

### Cell culture

293 human embryonic kidney cells [33, 34], 293T cells [35], HTX cells (a pseudodiploid subclone of HT-1080 human fibrosarcoma cells (ATCC CCL-121) [36]), IB3 immortalized human bronchial epithelial cells [18], and SSF-123 primary sheep skin fibroblasts (gift from William Osborne, University of Washington, Seattle), were grown in DMEM with 10% FBS in a 10% CO_2_/air atmosphere at 37 °C. The identity and human origin of the 293, HTX and IB3 cell lines was confirmed by short tandem repeat (STR) polymorphism analysis performed by the University of Arizona Genetics Core. The experimental STR data for these cell lines matched reference data for the 293 and HTX cell lines [37], and for the IB3 cell line [38].

### Detection of JSRV infection of human cells

Infectious JSRV was produced by calcium phosphate-mediated transfection of 293T cells with a plasmid that contains a JSRV provirus driven by a human cytomegalovirus (CMV) promoter, as previously described [39]. Briefly, cells were seeded at 3 × 10^6^ per 6-cm dish and 20 h later were transfected with 5 μg pCMV2-JSRV21 plasmid DNA per dish. The culture medium was changed 6 h after transfection and 24 h after the medium change, the virus-containing medium was collected, centrifuged at 2700 × *g* for 10 min to remove cell debris, and was filtered through 0.45-μm filters. Virus was stored at −80 °C.

For virus infection, cells were seeded in 6-well plates at 5 × 10^5^ per well (SSF, IB3 or HTX cells) or at 10^6^ per well (293 cells). 20 h later, the cells were treated with 400 μl of medium containing live JSRV, heat inactivated JSRV (65 °C for 15 min) or no virus, all in the presence of 5 μg/ml Polybrene (to facilitate infection). One day after treatment, the culture medium was replaced with fresh medium and the cells were cultured for an additional 2–7 days.

For analysis of integrated JSRV proviral DNA, culture medium was removed and the cells were washed twice with phosphate-buffered saline. Cells were detached using trypsin, were pelleted in 1.5 ml centrifuge tubes, and genomic DNA was extracted by using QIAamp DNA mini kits (QIAGEN, catalog #51306), according to the manufacturer’s instructions. Genomic DNA was quantified by using a Nanodrop ND-2000 UV–vis spectrophotometer (Thermo Fisher Scientific, Wilmington, DE). Then 250 ng genomic DNA was used as template to amplify JSRV specific fragments by using Dream Taq DNA polymerase (Thermo Fisher). Sense primer-I 5′TGGGAGCTCTTTGGCAGAAGCC3′ and reverse primer 5′CTGACGACTATGCGTTTGTCCC3′ were used to amplify the first round product, with expected size of 527 bp. Then 0.5 µl of first-round amplification products were used as templates to amplify the 2nd round products, by using sense primer-II 5′GCCTAGGACAAGTACCTAAGCTC3′ and reverse primer 5′CTGACGACTATGCGTTTGTCCC3′, with expected size of 502 bp. PCR products were resolved in 1% agarose gels with a 1 kb Plus DNA Ladder (Thermo Fisher) as a reference.

### Virus neutralization assay

The ability of serum to neutralize the infectivity of virus bearing the JSRV Env protein was measured. The virus used was a retroviral vector encoding placental alkaline phosphatase that was packaged using Gag-Pol proteins from Moloney murine leukemia virus and Env protein from JSRV (LAPSN(PJ4)) [2]. Briefly, approximately 150 infectious units of virus were mixed with various dilutions of untreated or heat-inactivated (56 °C for 30 min) serum in a total volume of 40 µl, and the mixtures were incubated at 20 °C (room temperature) for 30 min. The serum–virus mixtures were then added to HTX human fibrosarcoma cells in 6-well plates seeded the day before at 5 × 10^4^ cells per well. Infections were done in the presence of 4 µg/ml Polybrene. Two days after virus exposure, the cells were stained for alkaline phosphatase expression as described (Support Protocol 5) [40], and positive foci were counted. Antibody titers were expressed as the reciprocal of the highest dilution of serum that reduced the number of alkaline phosphatase-positive foci by ≥50%.

## Abbreviations

JSRV: jaagsiekte sheep retrovirus
OPA: ovine pulmonary adenocarcinoma
BAC: bronchioalveolar carcinoma
LCA: lung cancer
Env: retroviral envelope protein
SU: surface domain of a retroviral Env protein
Gag: retroviral group-specific antigen protein
MA: matrix domain of a retroviral Gag protein
Hyal2: hyaluronidase 2
